# Prioritizing the First Doses of SARS-CoV-2 Vaccine to Save the Elderly: The Case Study of Italy

**DOI:** 10.3389/fpubh.2021.684760

**Published:** 2021-07-14

**Authors:** Giuseppe Pontrelli, Giulio Cimini, Marco Roversi, Andrea Gabrielli, Gaetano Salina, Stefania Bernardi, Francesca Rocchi, Alessandra Simonetti, Carlo Giaquinto, Paolo Rossi, Francesco Sylos Labini

**Affiliations:** ^1^Academic Department of Pediatrics, Bambino Gesù Children's Hospital, Istituto di Ricovero e Cura a Carattere Scientifico, Rome, Italy; ^2^University of Tor Vergata, Rome, Italy; ^3^Physics Department and Istituto Nazionale di Fisica Nucleare, University of Tor Vergata, Rome, Italy; ^4^Enrico Fermi Research Center, Rome, Italy; ^5^Engineering Department, Roma Tre University, Rome, Italy; ^6^Department of Pediatrics, University of Padua, Padua, Italy

**Keywords:** COVID-19, SARS-CoV-2, vaccination, elderly, prioritizing 1st dose

## Abstract

SARS-CoV-2 is currently causing hundreds of deaths every day in European countries, mostly in not yet vaccinated elderly. Vaccine shortage poses relevant challenges to health authorities, called to act promptly with a scarcity of data. We modeled the mortality reduction of the elderly according to a schedule of mRNA SARS-CoV-2 vaccine that prioritized first dose administration. For the case study of Italy, we show an increase in protected individuals up to 53.4% and a decrease in deaths up to 19.8% in the cohort of over 80's compared with the standard vaccine recalls after 3 or 4 weeks. This model supports the adoption of vaccination campaigns that prioritize the administration of the first doses in the elderly.

## Introduction

Despite the restrictive measures adopted worldwide, the daily count of infections and deaths from COVID-19 remains high and unbearable. In western countries, the highest death toll has been paid by the elderly: in the case of Italy, of the ~102.010 deaths due to the pandemic, 62% were over 80-years-old, according to the bulletin of the Italian National Institute of Health (Istituto Superiore di Sanità, ISS), updated on March 17, 2021. The vaccination campaign has, therefore, prioritized this age group, whose immunization in Italy began on Monday, February 8, 2021 with difficulties due to various delays in the supply of the two approved mRNA vaccines (Pfizer/BNT Biotech and Moderna) initially dedicated to this cohort. The Italian strategic plan for anti-SARS-CoV-2/COVID-19 vaccination has been consequently adjusted several times (1).

The recommendation derived from registrative trials is that administration of the mRNA vaccines should be in two doses, spaced 3–4 weeks apart: the *efficacy* (protection assessed in clinical trials) of preventing symptomatic COVID-19 in clinical trials was 94.8 and 94.1% for Pfizer/BNT Biotech and Moderna, respectively (2, 3). However, when excluding cases of infection in the first 14 days after the first dose (the time needed for an effective immune response against the vaccine antigen), the same trial studies showed a good efficacy from the first dose alone: 92.6% (4) and 92.1% (3) for Pfizer/BNT Biotech and Moderna, respectively. A recent Israeli study also estimated first dose *effectiveness* (protection assessed in the real world, usually lower than efficacy) of 85% (95% CI 71–92) in reducing symptomatic COVID-19 cases (5). This data was confirmed in a study conducted in Scotland on healthcare workers (6). More recently, another study proved the efficacy of 57% (95% CI 50–63) from 14 to 20 days after the first dose and 66% (95% CI 57–73) from 21 to 27 days after the first dose (7). To date, the available data on vaccine efficacy against transmission is limited. However, an interim estimate of vaccine effectiveness of Pfizer/BNT Biotech and Moderna vaccines proved adjusted vaccine effectiveness against infection of 80% and 90%, respectively, 14 days after the first and second dose (8). What is well-known is that the vaccine reduces the symptomatic forms of COVID-19, thus also decreasing both the number of severely affected patients requiring admission to the ICU and deaths.

All these data refer to the short-term efficacy and effectiveness but show protection above the 50% threshold as considered by the European Medicines Agency (EMA) guidelines (9). Based on the epidemiological data and the scarcity of vaccine doses, the United Kingdom has adopted the strategy of postponing the administration of the second dose to 12 weeks after the first dose (10). The goal was self-evident: to protect as many people as possible as soon as possible, while waiting for a better supply of vaccines.

In Italy, the available doses of the two mRNA vaccines are much lower than those needed to immunize the entire population or even the over-80's in a short time. This situation is putting Italy, and other countries like the U.S., in front of a question similar to that faced by the United Kingdom: if the first objective is to save the greatest number of lives, why not delay the second doses until all high-risk subjects have been vaccinated with at least one dose?

Stanley Plotkin answers the question favorably (11), considering not only the apparent correlation between protection and low antibody levels after a single-dose administration of mRNA vaccine, as demonstrated in some studies, but also the relative efficacy of other vaccines, especially anti-hepatitis B, when administered at prolonged intervals (2, 12, 13). In addition, the study explains how memory B cells develop properly following the administration of mRNA vaccines, supporting the idea that further enhancement of antibody production is stimulated by a second dose of vaccine given up to 6 months after the first (14). Similarly, recent epidemiological modeling studies on SARS-CoV-2 showed the effectiveness of single dose vaccination strategy in containing the pandemic more rapidly (15). The debate is still ongoing among scientists and public health policymakers at both the national and international level (16, 17). The US CDC stated “There is no maximum interval between the first and second dose for either vaccine (Pfizer/BNT Biotech and Moderna). Therefore, if the second dose is administered >3 weeks after the first Pfizer-BioNTech vaccine dose or >1 month after the first Moderna vaccine dose, there is no need to restart the series” (18).

## Methods and Results

In this article, we provide a computation of the expected benefits to support the choice of the best vaccination strategy. We focused on the analysis of the Italian cohort of 4,442,048 people over 80-years-old, who are the first category to be vaccinated, according to the current National Vaccination Plan (1).

We formulated a simple effective model that estimates the number of protected individuals and deaths during the early phase of the vaccination campaign, by using data on vaccine efficacy from clinical trials and effectiveness from observational studies. We modeled a scenario in which the effectiveness of the 1st dose after 14 days is 0.80 ± 0.10 (5, 9) and a more conservative scenario with lower effectiveness of 0.60 ± 0.10 (6).

We considered a 7-week period (the model was originally conceived in the period February 10–March 31), with a varying rate of weekly vaccine administrations. We counted protected individuals at week 9 (i.e., April 14) and deaths from week 3 to week 9 included (i.e., February 24 to April 14), discounting the 2-weeks period required for coverage activation. Protected individuals in a given week were obtained as the total individuals vaccinated with 1 or 2 doses at least 2 weeks earlier, each modulated for the effective coverage provided by the vaccine. Deaths at a given week were then obtained through the weekly mortality parameter applied to the susceptible population (the cohort) after subtracting the protected individuals. For model parameters and mathematical details, please refer to the section below.

The vaccination campaign for over-80's and fragile individuals was carried out using preferably mRNA vaccines (Pfizer/BNT Biotech and Moderna). On February 22, the AstraZeneca vaccine was also indicated by Italian authorities for people > 65 years; however, evidence on its efficacy on over-80's remains incomplete, planned supplies are late, and administration of this vaccine has been further delayed by the temporary suspension set up by the EMA last March after the report of cases of cerebral vein thrombosis occurring after the vaccination. All these factors resulted in a delay in implementing the vaccination using the AstraZeneca vaccine in the cohort of over-80's.

**Figure 1:**
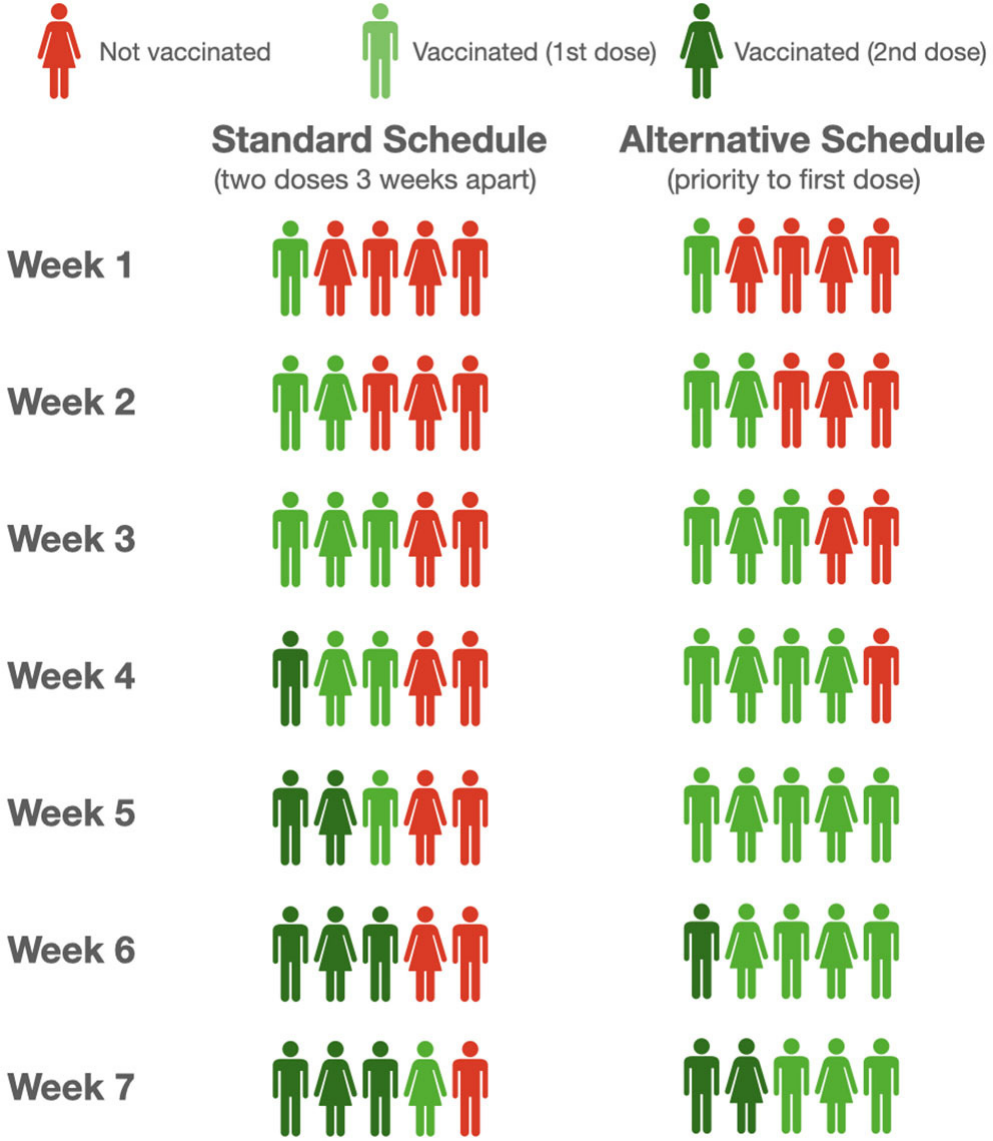
Schematic illustration of standard and alternative schedule of vaccination.

We considered two schedules of vaccine administration: a standard schedule of vaccine recall after 3 and 4 weeks, respectively, for Pfizer/BNT Biotech and Moderna, and an alternative schedule in which administration of the second dose begins after the whole cohort has received the first dose. In Figure 1, we report the pattern of vaccinated individuals under these two schedules over a 7 weeks period for only the Pfizer/BNT Biotech vaccine. The first 3 weeks are the same for both schedules. Afterward, under the standard schedule, the available doses will be reserved and used for the second vaccination of subjects who already been vaccinated, to the detriment of other individuals over 80-years-old not yet vaccinated, who will have to wait while remaining at risk.

**Figure 2:**
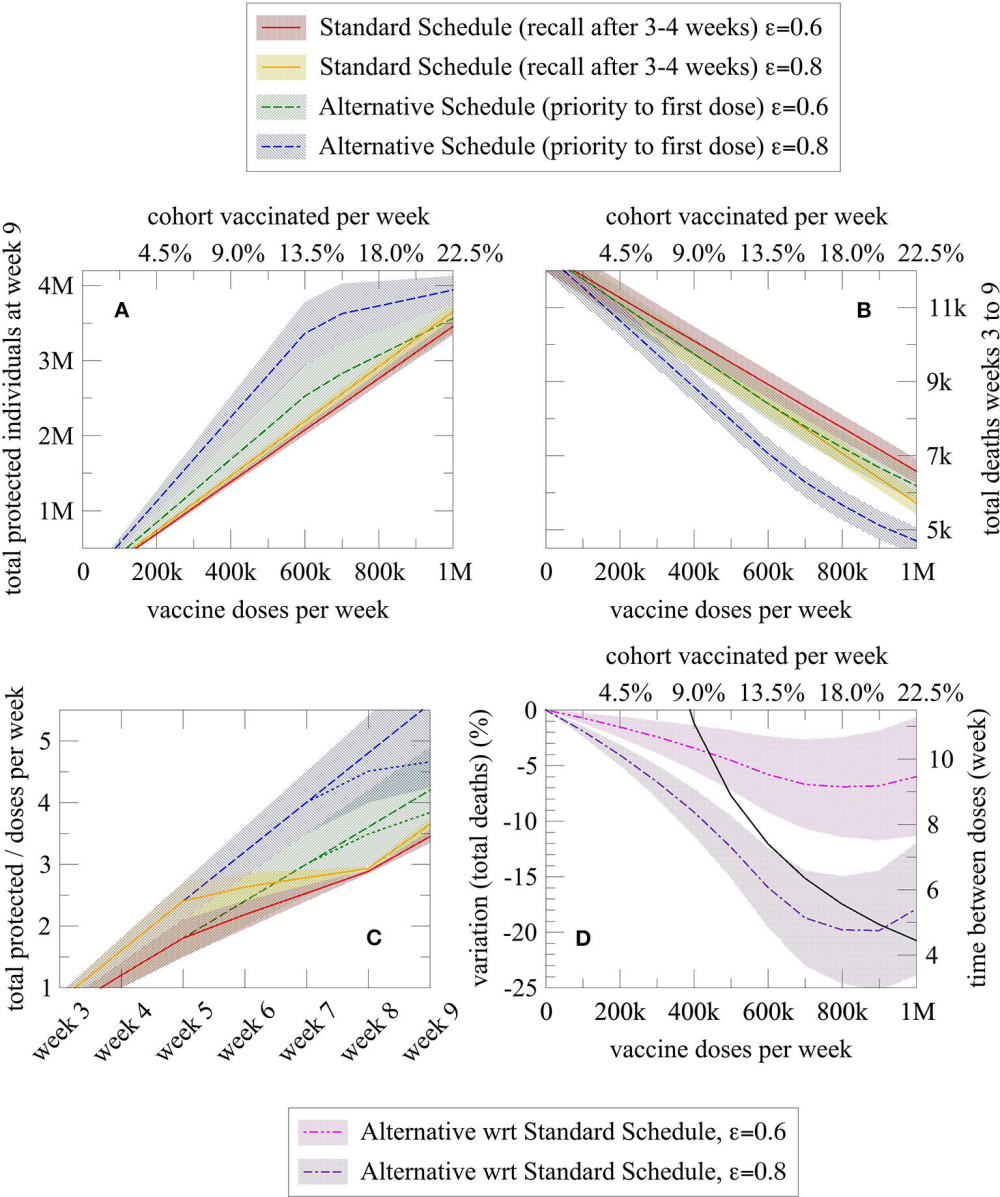
Graphical representation of the number of protected and dead individuals as a function of different variables, for a given vaccination schedule (alternative vs. standard). Each schedule is represented by two lines, one for vaccine effectiveness of 80% (blue for alternative schedule and orange for the standard schedule) and one for vaccine effectiveness of 60% (green for alternative schedule and red for the standard schedule). **(A)** Total protected individuals at week 9 and **(B)** Total deaths from week 3 to 9 as a function of the number of doses administered per week (or equally expressed as the percentage of the cohort vaccinated each week); **(C)** Efficiency index (= protected individuals per doses administered over time) of the alternative and standard schedule as a function of the elapsed weeks (dashed sublines indicated a higher number of doses administered, namely 800,000 per week); and **(D)** Death reduction with the alternative schedule (purple line for an 80% vaccine effectiveness and pink line for a 60% vaccine effectiveness) compared with the standard schedule, and effective week in which administration of second doses begins in the alternative schedule after the entire cohort has received the first dose of vaccine (black line). In all plots, the shaded region denotes the confidence interval derived from the uncertainty associated with the effectiveness of the first dose and mortality rate.

Consider, for example, a scenario with 800,000 doses (about 18% of the cohort) of mRNA vaccine was administered each week in the considered cohort. Considering the latency of 2 weeks necessary to evoke an efficient immune response (and assuming a first dose effectiveness of 80%), with this strategy a total of 2,920,000 people will be protected at week 9 according to the model. If we assume a constant weekly mortality rate of ~4 fatalities per 10,000 people in this age group [update as of March 10, 2021 (18)], there will be 7,061 deaths in the same cohort from week 3 to week 9. Conversely, following the alternative schedule (i.e., the strategy of prioritizing first doses to all individuals of the cohort before moving on to second doses), 3,727,330 people will be protected and a total of 5,664 deaths in the cohort are expected in the same time interval, i.e., a 27.6% increase of the protected and a 19.8% reduction of deaths, which notably is independent of the assumed mortality rate. The benefit is evident even when considering vaccine effectiveness of 60% and a different number of weekly vaccinations (refer to Table 1 and Figure 2), the simple reason being the number of protected growing at a very different pace when the first doses or the second doses are administered.

**Table 1:** Comparison between standard and alternative schedule.

		**Standard schedule: recall after 3-4 weeks**	**Alternative schedule: priority to first dose**
Doses per week		400,000 (~9.0% of cohort)	
Time between doses		3–4 weeks	11 weeks
1st dose effectiveness: 0.8	Protected at 14/04	1,460,000	2,240,000 (+53.4%)
	Deaths 24/2–14/4	9,750	8,854 (−9.2%)
1st dose effectiveness: 0.6	Protected at 14/04	1,380,000	1,680,000 (+21.7%)
	Deaths 24/2–14/4	10,094	9,750 (−3.4%)
Doses per week		600,000 (~13.5% of cohort)	
Time between doses		3–4 weeks	7.5 weeks
1st dose effectiveness: 0.8	Protected at 14/04	2,190,000	3,360,000 (+53.4%)
	Deaths 24/2–14/4	8,406	7,062 (−16.0%)
1st dose effectiveness: 0.6	Protected at 14/04	2,070,000	2,520,000 (+21.7%)
	Deaths 24/2–14/4	8,923	8,406 (−5.8%)
Doses per week		800.000 (~18.0% of cohort)	
Time between doses		3–4 weeks	5.5 weeks
1st dose effectiveness: 0.8	Protected at 14/04	2,920,000	3,727,330 (+27.6%)
	Deaths 24/2–14/4	7,061	5,664 (−19.8%)
1st dose effectiveness: 0.6	Protected at 14/04	2,760,000	3,070,512 (+11.2%)
	Deaths 24/2–14/4	7,751	7,213 (−6.9%)

As shown in Figures 2A,B, the alternative schedule increases protection and reduces the death in many more individuals than the standard one. The alternative schedule converges to the standard one, in terms of the number of protected individuals, only for about 1 million weekly administrations in this cohort, an extremely high and improbable number given the actual availability of doses; however, the advantage of the alternative schedule on the number of deaths is quite large at any time, because of the “advantage” in protection cumulated in the period covered by the model [see plot (C)]. Notably, in the plot (C), the number of protected individuals increases much more slowly over time with the standard schedule (red and orange line), plateauing at week 5 because second doses are being administered at that time. Instead, under the alternative schedule (blue and green lines), protected individuals increase linearly in time, with a possible bending when the number of weekly doses is so large that second doses administration start before week 7, as indicated by the black curve in the plot (D). Additionally, the plot (D) shows the sharp reduction in deaths with the alternative schedule, for both an 80% (purple line) and 60% (pink line) first dose vaccine effectiveness.

In addition, it is expected that the increase in protected individuals achieved by the alternative schedule would also reduce the number of hospitalizations in the ICU and in other inpatient units, thus alleviating the pressure on health structures and helping restore routine activities. Unfortunately, these indicators by age group are not reported in detail in Italian public reports as are the number of deaths, thus chosen as the study endpoint, however, they are estimated to be substantially higher (18).

It should also be noted that the strategy of temporarily postponing the second dose could be applied to other cohorts identified by the vaccination plan as priorities, for example extremely vulnerable individuals, as patients with chronic conditions that pose additional risk of death if infected, to obtain additional benefits, including patients in pediatric age, as Pfizer vaccine is authorized over 16 years of age. This benefit becomes even more evident in case of vaccine shortage, as mRNA vaccines would be then allocated to the fragile population they are meant for, independently from age, when applying this model. This study could be also implemented for other, younger ages and for estimating the impact on other important outcomes, such as ICU and hospital accesses.

Furthermore, as soon as vaccine supplies increase, the time interval between the two doses in the alternative schedule would be shortened, down to the recall times recommended by registrative trials. In any case, in the alternative schedule, the interval between the first and second doses is limited if the number of weekly vaccine administrations is high (<7.5 weeks if more than 600,000).

It may be argued that a delayed second dose facilitates the emergence of vaccine-resistant variants of the virus; however, the available data show that vaccines appear protective on variants now circulating in Europe, and the risk of this emergence is counterbalanced by the advantages of reducing viral circulation, by making more people non-susceptible to the virus in a shorter amount of time (6, 19–24).

## Conclusion

Many countries are facing high mortality caused by the circulation of SARS-CoV-2 among the elderly, who are not yet vaccinated. By prioritizing first dose administration, we estimated up to a 19.8% decrease in deaths in the cohort of over 80's, in case of effectiveness after the first dose of 80%, and a 6.9% decrease under the worst scenario of the effectiveness of 60%. This study has some limits. First, the data on vaccine efficacy was mainly derived from the total population, while this study focused on a cohort, namely the elderly, where the vaccination could be less effective. We proposed scenario 2 with low first dose effectiveness of 0.60 ± 0.10 to adjust for this aspect while still showing the usefulness of this model. Second, available data on efficacy and effectiveness mainly refer to a shorter time frame after the first dose, 4 weeks at most. In this model, we assumed a non-significant reduction of vaccine effectiveness at 11 weeks at most, as such a period may be required in case of vaccine shortages. Third, we do not embed this vaccination campaign into a formal susceptible-infected-recovered (SIR) based epidemiological model, as we assume the same virus circulation in the short time window considered when only the elderly are vaccinated. Such an embedding is a challenging task that would be likely be required to model longer time horizons and a population-wide vaccination coverage (25). Finally, we remark that this model focuses on the Italian scenario, which we consider as representative of other countries facing similar conditions in terms of infection rate, mortality, and vaccine supply, namely, the key parameters considered in this model. Overall, these findings suggest considering the vaccination option of prioritizing first doses in the elderly until the vaccine supplies are adequate.

### Model Formulation

*Parameters*:

- η = 0.95 (2, 3): effectiveness of second dose after 2 weeks
- ξ: effectiveness of first dose after 2 weeks:
  ∘ Scenario 1: 0.80 ± 0.10 (5)
  ∘ Scenario 2: 0.60 ± 0.10 (6)
- *v*: vaccine doses administered weekly (^*^)
- *z*_*P*_ ≃ 0.87 and *z*_*M*_ ≃ 0.13: ratio of available Pfizer/BNT Biotech and Moderna vaccines (1)
- *N* = 4,442,048 : cohort over 80 years of age
- μ = 0.0004 ± 0.0000415 (18) : weekly mortality rate (number of deaths on susceptible individuals) in over 80's in the week from 3 to 10 March 2021 in Italy

*Standard schedule:*

- Recall week: *T*_*P*_ = 3 (Pfizer/BNT Biotech) and *T*_*M*_ = 4 (Moderna)
- ${V_{1}}^{X}\left(t\right)$ and ${V_{2}}^{X}\left(t\right)$: vaccinated individuals with one or two doses of vaccine *X* = {*P, M*} at week *t*:
  ∘ ${V_{1}}^{X}\left(t\right)=vz_{X}t$ and ${V_{2}}^{X}\left(t\right)=0$ if *t* ≤ *T*_*x*_
  ∘ ${V_{1}}^{X}\left(t\right)=vz_{X}\left(2T_{X}-t\right)$ and ${V_{2}}^{X}\left(t\right)=vz_{X}\left(t-T_{X}\right)$ if *T*_*X*_ ≤ *t* < 2*T*_*x*_
  ∘ ${V_{1}}^{X}\left(t\right)=vz_{X}\left(t-2T_{X}\right)$ and ${V_{2}}^{X}\left(t\right)=vz_{X}T_{X}$ if *t* > 2*T*_*x*_
  Total vaccinated individuals with one or two doses: $V_{1}\left(t\right)={V_{1}}^{P}\left(t\right)+{V_{1}}^{M}\left(t\right)$ and $V_{2}\left(t\right)={V_{2}}^{P}\left(t\right)+\;{V_{2}}^{M}\left(t\right)$

*Alternative schedule:*

- Recall week: T^*^ = *N*/*v* (for both Pfizer/BNT Biotech and Moderna)
- *V*_1_(*t*) and *V*_2_(*t*): vaccinated individuals with one or two doses of vaccine (either {*P, M*}) at week *t* (expressions valid for *t* ≤ 2T^*^):
  ∘ *V*_1_(*t*) = min [*vt, N*] − *V*_2_(*t*)
  ∘ *V*_2_(*t*) = max [*vt, N*] − *N*

*Protected and deaths:*

- Protected individuals at week *t*: Π(*t*) = *V*_1_(*t*−2) ξ+*V*_2_(*t*−2) η
- Deaths at week *t*: *M*(*t*) ≃ μ [*N* − Π(*t*)]

(^*^) For Italy, using the full supply of vaccines for the first quarter of 2021 in the 4 weeks of the model would result in slightly over 1 million doses administered per week.

## Author Contributions

GP, GC, MR, AG, GS, and FS conceptualized, designed the article, and verified data. GP collected data. GP and GC analyzed data. GC created the tables and graphs. GP, GC, and MR drafted and wrote the manuscript. SB, FR, AS, CG, and PR reviewed the manuscript for important intellectual content. GP, GC, MR, AG, GS, SB, FR, AS, CG, PR, and FS revised the final manuscript. GP is guarantor for the article and expert in public health, clinical trials, and vaccine policy. MR is a medical doctor with expertise in scientific data collection, analysis, and scientific writing. GC, AG, GS, and FS are professors of Physics and experts in complex systems modeling. FR is a pharmacologist and expert in European regulatory affairs. All authors contributed to the article and approved the submitted version.

## Conflict of Interest

The authors declare that the research was conducted in the absence of any commercial or financial relationships that could be construed as a potential conflict of interest. The reviewer CR declared a shared affiliation, with no collaboration, with the authors, to the handling editor at the time of the review.
